# Echium Oil Reduces Plasma Triglycerides by Increasing Intravascular Lipolysis in apoB100-Only Low Density Lipoprotein (LDL) Receptor Knockout Mice

**DOI:** 10.3390/nu5072629

**Published:** 2013-07-12

**Authors:** Lolita M. Forrest, Christopher M. Lough, Soonkyu Chung, Elena Y. Boudyguina, Abraham K. Gebre, Thomas L. Smith, Perry L. Colvin, John S. Parks

**Affiliations:** 1 Department of Pathology/Section on Lipid Sciences, Wake Forest School of Medicine, Winston-Salem, NC 27157, USA; E-Mails: lolita_forrest@med.unc.edu (L.M.F.); chrislough@uky.edu (C.M.L.); skchung@ufl.edu (S.C.); eboudygu@wakehealth.edu (E.Y.B.); agebre@wakehealth.edu (A.K.G.); 2 Department of Orthopaedic Surgery, Wake Forest School of Medicine, Winston-Salem, NC 27157; USA; E-Mail: tsmith@wakehealth.edu; 3 Division of Geriatric Medicine and Gerontology, Johns Hopkins University School of Medicine, Baltimore, MD 21224, USA; E-Mail: pcolvin1@jhmi.edu; 4 Department of Biochemistry, Wake Forest School of Medicine, Winston-Salem, NC 27157, USA

**Keywords:** fish oil, PUFA, polyunsaturated fatty acids, *n*-3 fatty acids, omega-3 fatty acids, very low density lipoproteins, botanical oil, hepatic steatosis

## Abstract

Echium oil (EO), which is enriched in SDA (18:4 *n*-3), reduces plasma triglyceride (TG) concentrations in humans and mice. We compared mechanisms by which EO and fish oil (FO) reduce plasma TG concentrations in mildly hypertriglyceridemic male apoB100-only LDLrKO mice. Mice were fed one of three atherogenic diets containing 0.2% cholesterol and palm oil (PO; 20%), EO (10% EO + 10% PO), or FO (10% FO + 10% PO). Livers from PO- and EO-fed mice had similar TG and cholesteryl ester (CE) content, which was significantly higher than in FO-fed mice. Plasma TG secretion was reduced in FO *vs*. EO-fed mice. Plasma very low density lipoprotein (VLDL) particle size was ordered: PO (63 ± 4 nm) > EO (55 ± 3 nm) > FO (40 ± 2 nm). Post-heparin lipolytic activity was similar among groups, but TG hydrolysis by purified lipoprotein lipase was significantly greater for EO and FO VLDL compared to PO VLDL. Removal of VLDL tracer from plasma was marginally faster in EO *vs*. PO fed mice. Our results suggest that EO reduces plasma TG primarily through increased intravascular lipolysis of TG and VLDL clearance. Finally, EO may substitute for FO to reduce plasma TG concentrations, but not hepatic steatosis in this mouse model.

## 1. Introduction

Dysregulation of lipid metabolism results in several chronic diseases, including diabetes, obesity, and cardiovascular disease (CVD). Overwhelming evidence supports increased dietary consumption of *n*-3 PUFAs for CVD prevention and treatment [1,2,3]. Epidemiological studies first reported the beneficial effects of fish oil (FO) on plasma lipids and CVD of Greenland Eskimos compared to Danish residents [4,5,6]. The beneficial effects of FO are attributed to its *n*-3 fatty acid enrichment of eicosapentanoic acid (EPA, 20:5) and docosahexaenoic acid (DHA, 22:6). Since then, many studies have shown that dietary FO or formulations of EPA and DHA are cardioprotective [7,8,9,10,11], due to decreased inflammation, plasma lipid concentrations, endothelial cell activation, and cardiac arrhythmia [12,13,14,15]. The most consistent lipid effect of dietary FO is reduced triglyceride (TG) concentrations [16,17]. Although high plasma LDL cholesterol concentrations are associated with increased CVD risk, plasma TG may be an independent risk factor [18].

While the effectiveness of FO at reducing plasma TG concentrations is well established, the mechanisms behind this effect are unclear. *N*-3 PUFAs may regulate the expression of genes involved in lipogenesis, including SREBP1c and PPAR alpha [19,20,21,22]. Other studies in humans and rodents have suggested FO-mediated TG-lowering is a cumulative effect of increased fatty acid β-oxidation, reduced TG synthesis and secretion, and enhanced plasma TG-rich particle clearance [23,24,25].

Despite the well-documented beneficial effects of fish and FO supplements, they are not widely consumed by the US population. The recommended dietary intake ratio of *n*-6:*n*-3 PUFAs is 2.3:1, but North Americans consume a ratio closer to 10:1 [26,27]. Potential reasons for this fact include cost, personal preference, and FO supplement aftertaste. Approximately 90% of *n*-3 PUFA in North American diets comes from vegetable oil-derived α-linolenic acid (ALA) [26]. However, ALA is not sufficiently metabolized to longer-chain PUFAs, such as EPA and DHA, due to inefficient Δ-6 desaturation in mammals [28]. Therefore, finding a suitable alternative to FO to increase consumption of long chain *n*-3 PUFAs could considerably improve cardiovascular health.

Echium oil (EO), derived from *Echium plantagineum* seeds, may be an alternative to FO. Approximately 13% of the total fatty acids in EO is stearidonic acid (SDA; 18:4 *n*-3), the immediate product of ALA Δ-6 desaturation [29]. Hypertriglyceridemic patients given EO had decreased plasma TG concentrations and increased EPA enrichment in plasma and neutrophils, suggesting that SDA in EO was efficiently elongated and desaturated to EPA (19). We observed reduced plasma TG and hepatic lipogenic gene expression in mice after 8 weeks of EO supplementation [30].

In this study, we examined the effects of dietary EO and FO on mechanisms of VLDL production and catabolism in male apoB100-only LDLrKO mice. This model of atherosclerosis exhibits mildly elevated plasma TG levels [31]. The goal of this study was to determine whether the mechanisms of TG lowering were similar for EO and FO.

## 2. Experimental Section

### 2.1. Animals and Diets

Male apoB100-only mice were created by Dr. Steve Young [32]. They were then crossed with LDLrKO mice and used in a mixed C57BL/6 background (~93%) for previous studies [30,33]. Mice for this study were backcrossed 5–6 times with C57BL/6 LDLrKO mice and determined to be >99% in the C57BL/6 background by genome-wide screens using 134 single nucleotide polymorphisms that were polymorphic between the C57BL/6 and 129/SVEV strains and spaced approximately 20 Mb across the mouse genome. The animals were housed in a specific-pathogen free facility at Wake Forest School of Medicine. All experimental procedures were approved by the Institutional Animal Care and Use Committee.

At 8 weeks of age, mice were randomly assigned to one of three experimental diets: palm oil (PO), fish oil (FO), or echium oil (EO). Each diet contained 0.2% cholesterol, 0.2% FO (to prevent essential fatty acid deficiency), and 10% of calories from PO, with an additional 10% of calories from either PO, FO, or EO, for a total of 20% calories as fat. Complete diet [8] and fatty acid compositions [30] have been previously described. All experiments were performed on mice after 4–8 weeks of diet consumption except for liver lipid and VLDL compositional analyses, which were conducted in mice fed for 16 weeks.

### 2.2. Plasma Lipid Analysis

All blood samples were collected via tail bleeding into 75 µL heparinized capillary tubes from 4 h fasted mice. Plasma was isolated by centrifugation of blood at 12,000 rpm (4 °C) for 10 min and used immediately or stored at −20 °C for later use. Plasma cholesterol (Wako) and TG (Roche) were determined by enzymatic analysis as described previously [34]. ApoB concentrations were determined by SDS-PAGE and Western blotting using 2 µL of whole plasma. Separated proteins were then transferred to a nitrocellulose membrane and apoB visualized using a 1:5000 dilution of apoB100 goat anti-human primary antibody (Biodesign), 1:5000 α-goat IgG HRP secondary antibody (Sigma), and chemiluminescence substrate (Thermo Scientific). ApoB band intensity was quantified using a Fuji Film Cold Camera and Multi-Gauge Software. Plasma lipids were extracted using the Bligh-Dyer method [35] for fatty acid analysis. Lipids were fractionated into phospholipid (PL), TG, and cholesteryl ester (CE) bands by thin-layer chromatography using a neutral solvent system. Each fraction was methylated and analyzed by gas-liquid chromatography for fatty acid content, as previously described [34].

### 2.3. RNA Analysis

TRIzol reagent was used to isolate total RNA from livers of mice after 16 weeks of experimental diet feeding. One ug of RNA was converted to cDNA and 20 ng of cDNA was used to perform quantitative real-time PCR using SYBR Green PCR master mix (Applied Biosystems). GAPDH was used as the housekeeping gene and the results were evaluated using the 2^−ΔΔCT^ method [36].

### 2.4. VLDL Characterization

After 16 weeks of experimental diet feeding, mouse plasma VLDL was isolated to determine size, lipid and protein composition, and particle number. VLDL was isolated from plasma by ultracentrifugation at 100,000 rpm for 4 h at 15 °C at a density of 1.006. The VLDL fraction was removed by tube slicing and re-isolated to minimize albumin contamination. VLDL was used immediately or stored at −20 °C for later use. VLDL total cholesterol, free cholesterol, and TG concentrations were determined by colorimetric enzymatic assays [34]. Cholesteryl ester was calculated as [total cholesterol (TC) − free cholesterol (FC)] × 1.67 (to correct for loss of fatty acid after saponification). PL was quantified by a PL phosphorus assay [37]. Protein concentrations were determined by micro BCA assay. The surface-to-core ratio of VLDL constituents was calculated from the measured chemical constituents (surface constituents: FC + PL + protein; core constituents: CE + TG) and used as a surrogate measurement of VLDL particle size. Two µL of isolated VLDL dissolved in 43 µL saline was used to determine VLDL size by light scatter analysis using a Zetasizer Nano Series light scatter instrument (Malvern Instruments, Westborough, MA, USA). The resulting profile was analyzed using Disperson Technology Software 4.2 (Malvern Instruments) and VLDL size was quantified as the primary peak in the analysis by volume.

### 2.5. Hepatic Lipid Analysis

Livers from mice fed for 16 weeks were used to conduct liver lipid analysis. Lipids were extracted using 2:1 chloroform:methanol, 1% Triton X-100 in chloroform was added, and the mixture was dried under a stream of N_2_ gas at 60 °C and resuspended in water for a 1:2 final ratio of dH_2_O:Triton X-100. TG, TC, FC, and CE content were then quantified by enzymatic analysis as described above.

### 2.6. Determination of Plasma Post-Heparin Lipase Activity

Plasma lipoprotein lipase (LPL) and hepatic lipase (HL) activities were quantified using post-heparin plasma as a source of enzyme and a micellar artificial substrate. Briefly, animals were fasted for 4 h before tail vein injection of 100 units/kg (180 mg/kg) heparin (sodium salt dissolved in saline) to release capillary-bound lipases. Ten minutes post-injection, a blood sample was taken and plasma was separated by low-speed centrifugation. The substrate for total lipase activity was prepared and the assay performed as previously described [38,39], except 10 µL plasma was used as the enzyme source and 1 µg apolipoprotein CII (Sigma) was added to each reaction to activate LPL. Hepatic lipase activity was determined by adding 95 µL 4 M NaCl to inhibit lipoprotein lipase. Lipoprotein lipase activity was calculated as the difference between the total lipase activity and HL activity.

### 2.7. VLDL Lipolysis by LPL

To determine the extent to which diets affected VLDL TG hydrolysis, we isolated plasma VLDL from mice in the three diet groups and incubated the VLDL particles with LPL *in vitro*. VLDL (2 µg TG) was added to a microtiter plate and adjusted to equal volumes with water. Twenty-five ng of LPL (Sigma) and 1 µg of apoCII (as activator, Sigma) were added to initiate the hydrolysis. Duplicate samples minus apoCII were used to subtract background lipolysis. The samples were incubated at 37 °C for 1 h, after which release of NEFA was measured using a modification of the NEFA C method (Wako). NEFA C reagents were added per the manufacturer’s instructions and the amount of free fatty acids released was measured by a colorimetric reaction, using oleic acid (OA) standards. Pilot studies were performed to establish that these conditions resulted in a linear increase in fatty acid hydrolysis as a function of increasing VLDL TG substrate. Lipase activity was calculated as µmol FA released/h/mL.

### 2.8. TG Synthesis Rate Analysis

Hepatic secretion rate was determined in mice fed the experimental diets for 4–6 weeks. After a 4 h fast, mice were anesthetized with ketamine/xylazine and a baseline retro-orbital blood sample was taken. Then, mice were injected retro-orbitally with an equal volume of Triton WR 1339 (500 mg/kg mouse) and 0.9% NaCl [40]. Blood samples were taken at 45, 90, and 180 min post-injection. Plasma TG concentrations were measured by enzymatic analysis as described above. Hepatic secretion rates were determined by calculating the slope of TG values over the time course (0 to 180 min) using GraphPad Prism software.

### 2.9. Turnover Studies

One PO-fed and two EO-fed mice, fed their respective diets for at least 16 weeks, were used as donor mice. Mice were terminally bled by cardiac puncture and plasma was collected as previously described. Plasma from the two EO-fed mice was pooled. VLDL was then isolated from both samples as previously described. VLDL (150 µg protein) was radioiodinated with 50 µCi of radioactive iodine (PO-VLDL with ^125^I, and EO-VLDL with ^131^I) using the ICl method [41] and desalted on a 10 mL Bio-Rad-Pac10 disposable chromatography column. VLDL was then dialyzed in 12 mM phosphate buffer containing 124 mM NaI (pH 7.4) and radiolabel, and protein was measured to calculate specific activity (*i.e.*, dpm/µg protein). 550,000 cpm of each VLDL dose was combined and injected into PO-fed and EO-fed recipient mice via the jugular vein. Blood was collected 5 min, 30 min, 1, 2, 4, 6, 8, and 24 h post-injection. A multi-compartmental model (SAAM II program) was used to estimate the fractional catabolic rate (FCR), as described previously [42]. Plasma was separated as described above and apoB radioactivity was measured after isopropanol precipitation by gamma counting. Die-away curves were plotted as % of 5 min plasma radioactivity. Plasma samples were also separated by FPLC to determine the amount of radiolabel remaining as VLDL 30 min, 3 h, and 8 h post-injection.

### 2.10. Statistical Analysis

All data are presented as mean ± SEM. Differences among the 3 diet groups were analyzed by one-way ANOVA (*p* < 0.05) using GraphPad Prism software. Individual diet differences were identified using Tukey’s post-test analysis.

## 3. Results

### 3.1. Echium Oil Reduces Plasma Triglyceride Concentrations

**Figure 1:**
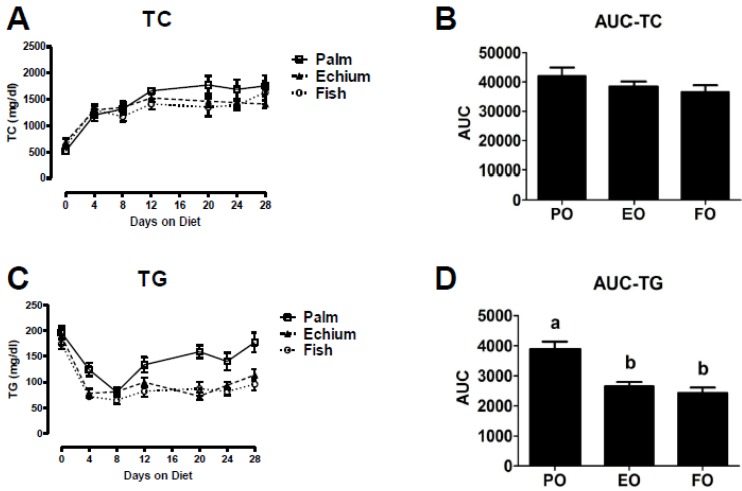
Plasma cholesterol and triglyceride (TG). After a 4 h fast, blood was collected via tail bleeding into 75 µL heparinized capillary tubes. Plasma was isolated from blood by centrifugation at 12,000 rpm at 4 °C. Animals were bled at baseline (0 days), and after 4, 8, 12, 20, 24, and 28 days on diet. Plasma total cholesterol (TC, Wako) and triglyceride (TG, Roche) concentrations were determined by enzymatic assays. Area under the curve (AUC) for each animal was calculated and TC and TG differences among the three diet groups were determined by one-way ANOVA using GraphPad Prism software. TC (*n* = palm oil (PO)-6, echium oil (EO)-11, and fish oil (FO)-7) (**A**), area under the curve for TC (**B**), TG (*n* = PO-7, EO-12, and FO-8) (**C**) and area under the curve for TG (**D**). Values represent mean ± S.E.M. and different letters represent a significant difference (*p* < 0.0001).

In this study, mice were switched from chow to the experimental diets at eight weeks of age and total plasma cholesterol (TPC) and TG concentrations were examined at earlier times after experimental diet initiation. Four days after diet initiation, TPC concentrations increased nearly three-fold for all three diet groups compared to the chow baseline (*i.e.*, zero time point). Thereafter, TPC increased with time, but differences in values were not seen over the 28-day period (Figure 1A,B, AUC = area under the TPC *vs*. time curve). Plasma TG concentrations decreased 50% within 4 days of experimental diet feeding and remained unchanged for the EO and FO groups, but increased to significantly higher values for the PO group (Figure 1C,D). These data show the rapid hypotriglyceridemic effect of the *n*-3 diets in our model. Furthermore, in a separate cohort fed the experimental diets for 16 weeks, body and liver weights were similar throughout the study. Additionally, after 16 weeks, plasma PL, TG, and CE fatty acid compositions reflected that of the experimental diets (Supplemental Figure S1), similar to our previous 8-week study [30].

### 3.2. Echium Oil Alters VLDL Composition

**Figure 2:**
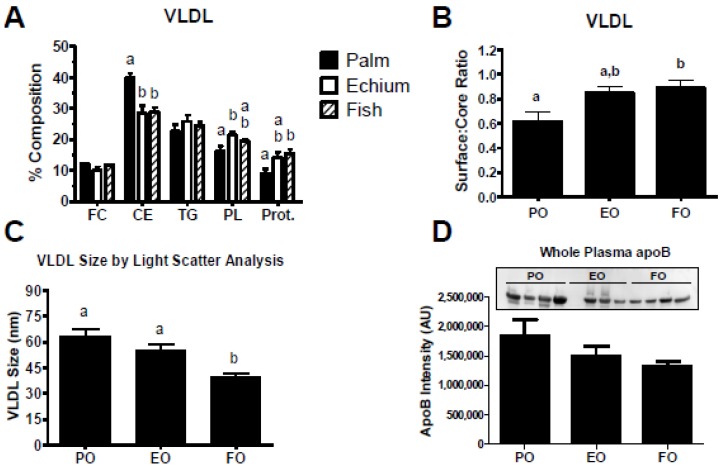
Plasma very low density lipoprotein (VLDL) compositional and size analysis. (**A**) VLDL from mice fed PO (*n* = 5), EO (*n* = 5), and FO (*n* = 9) were used for free cholesterol (FC), cholesteryl ester (CE), triglyceride (TG), phospholipid (PL), and protein quantification and percentage chemical composition was calculated. (**B**) VLDL surface to core ratio. (**C**) VLDL from animals fed PO (*n* = 12), EO (*n* = 15), and FO (*n* = 14) were isolated and particle diameter was measured by laser light scatter. (**D**) Whole plasma apoB concentration was analyzed by Western blot analysis. Plasma (2 µL) was fractionated by 4%–8% SDS PAGE from mice fed diets containing PO (*n* = 4), EO (*n* = 3), and FO (*n* = 4). ApoB was detected by Western blot analysis and chemiluminescent band intensity was measured using Multi Gauge software. Values are mean ± S.E.M. Values with different letters are significantly different (*p* < 0.05).

Since plasma TG levels were strikingly decreased for the two *n*-3 diet groups, a terminal blood sample was collected after 16 weeks of diet feeding to isolate plasma VLDL for measurement of chemical concentration and particle size. VLDL particles from EO and FO fed mice had significantly less CE and corresponding increases in PL and protein (Figure 2A). These compositional differences suggested a difference in VLDL particle size, since the *n*-3 groups had decreased core and increased surface constituents. Calculation of the surface-to-core ratio (*i.e.*, FC + PL + protein/CE + TG), an indirect measurement of VLDL particle size, suggested smaller VLDL particles in the EO and FO groups (*i.e.*, increase in ratio; Figure 2B). Further, analysis of VLDL particle size by laser light scatter revealed a tendency, though not significant, for decreased particle size for the EO and FO groups compared to the PO diet group, which was statistically significant for the FO *vs*. PO comparison (Figure 2C). Whole plasma apoB particle concentrations (measured by Western blot) tended to decrease in the EO and FO groups but did not reach statistical significance (Figure 2D).

### 3.3. Echium Oil does not Affect Liver Lipid Content and Gene Expression

**Figure 3:**
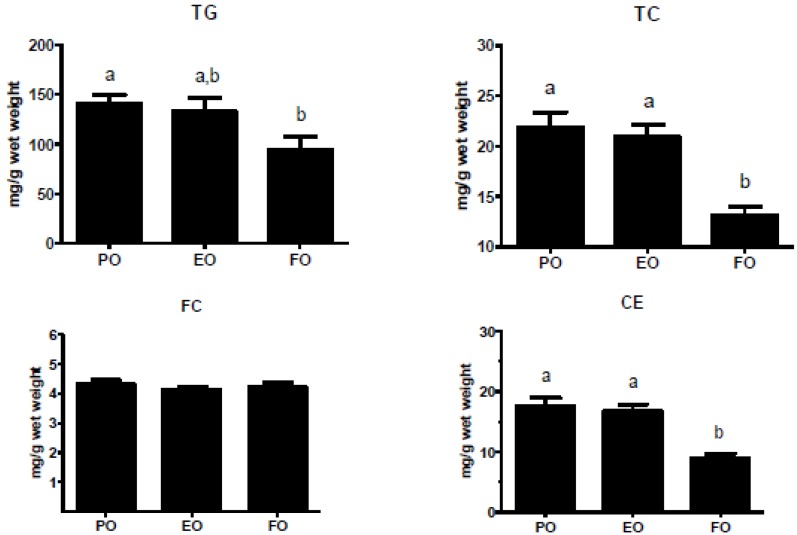
Liver lipid content. Mice were fed experimental diets containing PO, EO, or FO for 16 weeks before livers were harvested, lipids extracted, and lipid content was measured using enzymatic assays. The bars represent mean ± S.E.M. for PO (*n* = 16), EO (*n* = 13), and FO (*n* = 12). Values with different letters are significantly different (*p* < 0.05). TG, triglyceride; TC, total cholesterol; FC, free cholesterol; CE, cholesteryl ester.

**Figure 4:**
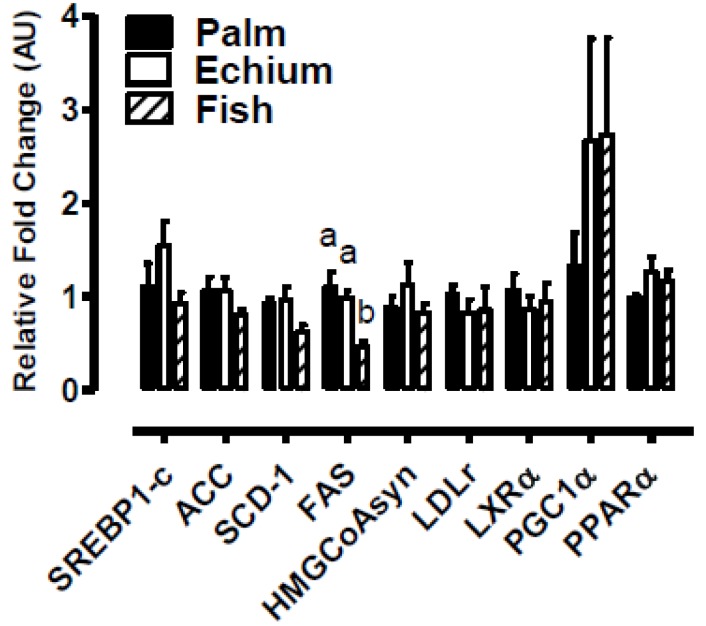
Hepatic gene expression. Mice were fed experimental diets containing PO, EO or FO for 16 weeks before livers were harvested for measurement of gene expression by quantitative real-time PCR. Liver RNA was isolated using TRIzol from individual mice and quantified for the indicated genes. Values represent mean ± S.E.M.; *n* = 3–5 per diet group. Values with different letters are significantly different from one another (*p* < 0.05) for each gene. SREBP1-c, sterol regulatory element binding protein 1-c; ACC, acetyl CoA carboxylase; SCD-1, stearoyl CoA desaturase-1; FAS, fatty acid synthase; HMGCoA synthase, hydroxymethylglutaryl CoA synthase; LDLr, low-density lipoprotein receptor; LXRα, liver X receptor α; PGC1α, peroxisome proliferator-activated receptor γ, coactivator 1α; PPARα, peroxisome proliferator-activated receptor α.

Previously, we found a trend towards decreased hepatic TG and CE content after 8 weeks of the EO diet compared to the PO diet [30]. However, after 16 weeks, hepatic lipid content in the EO group was more similar to that of the PO group than in our shorter study. FO-fed mice had significant decreases in hepatic TC and TG, while PL remained similar among all groups (Figure 3). Furthermore, genes involved in TG biosynthesis, such as SREBP1-c, ACC, SCD-1 and FAS, were expressed to a similar extent for EO- *vs*. PO-fed mice, whereas expression was reduced, in general, for FO-fed mice (Figure 4).

### 3.4. Echium Oil does not Affect liver TG Secretion Rate

**Figure 5:**
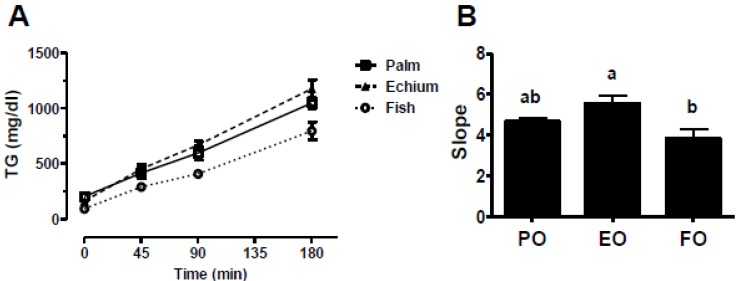
Hepatic TG secretion rate. (**A**) Mice fed PO, EO, or FO diets were fasted for 4 h before baseline TG values were determined by enzymatic analysis. Animals were injected retro-orbitally with Triton X-100 (500 mg/kg mouse) to block lipase activity. Hepatic TG secretion rate was calculated by measuring TG accumulation in plasma 45, 90, and 180 min post-injection. (**B**) Slopes of hepatic TG secretion shown in Panel **A**. Values represent mean ± S.E.M. Values with different letters are significantly different (*p* < 0.05).

One potential mechanism for reduced plasma TG concentrations in EO-fed mice is decreased hepatic VLDL TG secretion into plasma. To test this possibility, mice fed PO, EO, and FO diets for 4–6 weeks were fasted for 4 h and injected with Triton WR 1339 (500 mg/kg mouse) to inhibit plasma lipase activity [40]. The accumulation of TG in plasma was subsequently measured over 3 h (Figure 5). The accumulation rate of plasma TG was significantly less for mice fed FO compared to those fed EO, whereas the accumulation for PO-fed animals was intermediate. Thus, hepatic VLDL TG secretion could not account for the reduced plasma TG concentration for EO- *vs*. PO-fed mice.

### 3.5. Echium Oil-Derived VLDL Particles Are More Susceptible to Hydrolysis

**Figure 6:**
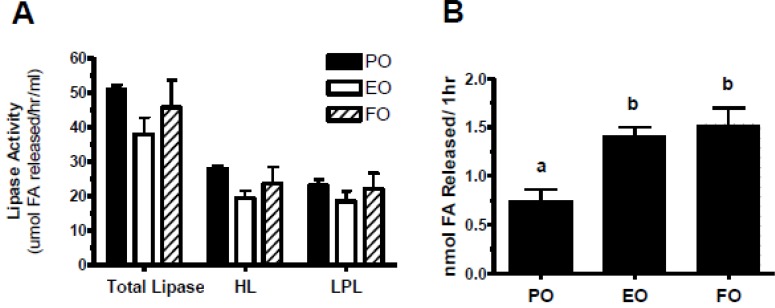
Post-heparin plasma lipase activity. (**A**) Total lipase, hepatic lipase (HL), and lipoprotein lipase (LPL) activities in plasma of mice after 28 days of PO, EO, or FO diets (*n* = 4, *n* = 6, *n* = 4, respectively). After a 4 h fast, mice were injected with 100 units/kg (180 mg/kg) heparin sodium salt (Sigma) dissolved in saline. 10 min post-injection, blood was collected and plasma separated as described previously. Triolein (Sigma) was used as a substrate. See Materials and Methods for details on substrate preparation. Post-heparin plasma was combined with the substrate and apoCII (Sigma) as a co-activator. HL activity was assayed by salt inhibition of LPL and LPL activity was calculated as total lipase activity minus HL activity. (**B**) VLDL lipolysis by purified LPL. 2 µg of VLDL TG from each diet group (PO, *n* = 7; EO, *n* = 11; FO, *n* = 3) were incubated with 25 ng of LPL (Sigma) for 1 h at 37 °C. Total NEFA released were measured by colorimetric analysis using a NEFA assay (Wako). The bars represent mean ± S.E.M. Values with different letters are significantly different (*p* < 0.05).

Lowered TG concentrations that accompany FO consumption may be mediated by increased lipolysis [43,44]. However, in hypertriglyceridemic human subjects 4 weeks of FO decreased plasma TG without affecting LPL and HL activity [45]. To determine whether the reduced plasma TG concentrations in EO-fed mice were due to increased TG lipolysis, we measured post-heparin lipase activity using a standard TG micellar substrate to test for increased lipase activity. We isolated plasma VLDL and measured TG lipolysis using purified LPL to test for increased lipolysis of TG with EO VLDL. As shown in Figure 6A, HL and LPL activities were similar among the three diet groups, suggesting that decreased plasma TG in EO-fed mice was not due to increased lipase activity. However, incubation of isolated VLDL with purified LPL resulted in significantly increased levels of VLDL TG lipolysis, measured as free fatty acid release, for VLDL isolated from EO- and FO-fed mice compared to PO-fed mice (Figure 6B).

### 3.6. Echium Oil Has Minimal Impact on Plasma VLDL Particle Turnover

**Figure 7:**
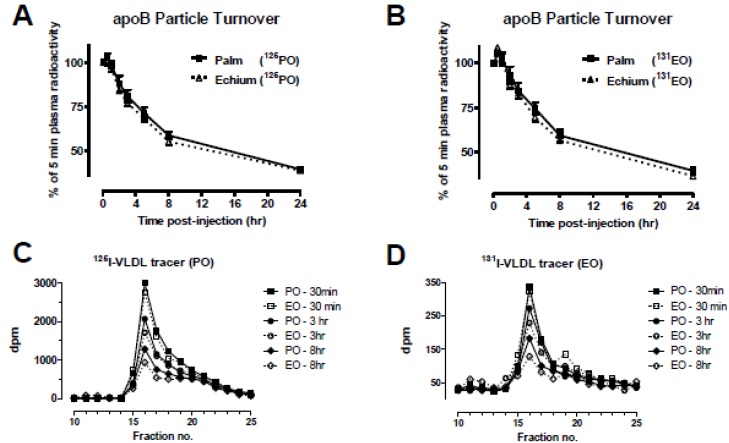
VLDL particle turnover. PO and EO recipient mice were injected with a mixture of ^125^I-VLDL from PO-fed, and ^131^I-VLDL from EO-fed donor mice via the jugular vein. (**A**) The rate of removal of VLDL tracer from PO-fed donor mice. Plasma was collected 5 min, 30 min, 1 h, 2 h, 4 h, 8 h, and 24 h after tracer injection and apoB radioactivity was measured after isopropanol precipitation. Data are presented as % of 5 min radioactivity remaining in plasma. (**B**) The rate of removal of VLDL tracer from EO-fed donor mice. See A for details. (**C**) Elution profile of PO VLDL tracer in PO and EO recipient mouse plasma. Plasma samples were separated by FPLC to determine the amount of radiolabel remaining as VLDL 30 min, 3 h, and 8 h post-injection. (**D**) Elution profile of EO VLDL tracer in PO and EO recipient mouse plasma. See C for details.

Since EO VLDL particles were lipolyzed to a greater extent by purified LPL compared to PO VLDL, we investigated whether EO VLDL particles had increased removal rates from plasma. After a 4 h fast, PO- and EO-fed recipient mice were injected with a mixture of ^125^I-radiolabeled PO VLDL and ^131^I-radiolabeled EO VLDL in a cross-over design. Plasma samples were taken over 24 h and apoB radiolabel was quantified after isopropanol precipitation of apoB from plasma [46]. Plasma die-away curves for VLDL apoB are shown in Figure 7. Because recipient mice lack active LDL receptors, VLDL removal from plasma was slow relative to wild-type mice [47]. Plasma die-away curves were similar for both diet groups regardless of the source of VLDL tracer (Figure 7A,B). Several plasma time points taken after VLDL tracer injection were size-fractionated by FPLC to determine whether the radiolabel remained in the VLDL fraction or was converted to LDL-sized particles. Most of the VLDL tracer remained in the VLDL size range, suggesting minimal conversion of tracer to LDL particles during the first 8 h of the turnover study (Figure 7C,D). There was also a tendency towards reduced radiolabel in plasma at 3 and 8 h compared with the 30 min sample for EO recipients, regardless of the source of the VLDL tracer (Figure 7C,D). However, fractional catabolic rate (pools/day) for both VLDL tracers was similar in PO (0.603 ± 0.123; *n* = 5) and EO (0.783 ± 0.057; *n* = 5) recipient mice, suggesting a minimal effect of dietary fat type on VLDL catabolism in the absence of LDL receptors.

## 4. Discussion

Diets enriched in FO result in significantly decreased plasma TG concentrations in humans and animals. Previous studies have shown that EO, a botanical source of *n*-3 PUFAs, also results in significantly reduced plasma TG concentrations in humans and mice relative to oils devoid of *n*-3 PUFAs [30,48]. This study was designed to determine how EO mediates its plasma hypotriglyceridemic effects and whether they are similar to those of FO. Using a mildly hypertriglyceridemic mouse model, we observed similar reductions in plasma TG concentrations for EO and FO relative to PO. However, upon evaluation of hepatic VLDL TG production rates, plasma apoB concentrations, VLDL TG lipolysis by LPL, post-heparin plasma lipolytic activity, and VLDL particle turnover, we discovered that FO exerts its hypotriglyceridemic effect by decreasing hepatic VLDL TG production and increasing plasma VLDL TG lipolysis, whereas only the latter mechanism was observed for EO-fed mice. Our results suggest that EO may be a suitable botanical alternative to FO for *n*-3 PUFA enrichment and reduced plasma TG concentrations, but not hepatic steatosis, at least in this animal model.

One proposed mechanism for FO-mediated TG lowering is reduced TG synthesis and secretion into plasma. In humans, nonhuman primates, and other animal models, FO reduced VLDL-TG production and secretion [49,50,51,52]. Furthermore, FO-mediated TG reduction appears secondary to reduced fatty acid availability [53]. After 16 weeks of treatment, we found that hepatic TG content was significantly reduced with FO feeding (but not EO feeding) compared to PO. This finding suggests that, although both diets resulted in reduced plasma TG concentrations, the mechanisms involved may be different. Similar findings in hepatic TG secretion during our detergent block studies for the PO- and EO-fed groups also suggest unique TG-lowering mechanisms for PO and EO (Figure 5).

These findings are supported by relative increases in lipogenic gene expression in EO- *vs*. FO-fed mice. Sterol regulatory element binding protein 1c (SREBP1c) is a major target of PUFA control in the liver [54]. *N*-3 PUFAs decrease the expression of SREBP1c, which acts indirectly to induce lipogenesis by inducing transcription of several lipogenic genes, such as fatty acid synthase (FAS), acetyl CoA carboxylase (ACC), and stearoyl CoA desaturase-1 (SCD-1) [55,56,57]. We have previously shown in the apoB100-only LDLrKO mouse model that both EO and FO reduce hepatic SREBP1c, FAS, and SCD-1 expression after 8 weeks of feeding [30], but the effect was not maintained after 16 weeks of feeding. This suggests that prolonged EO feeding is sufficient to maintain significantly lower plasma TG concentrations compared to PO, even under conditions of apparent hepatic lipotoxicity.

Pan *et al.* suggested that PUFA peroxidation is involved in the regulation of apoB degradation [58]. ApoB alterations such as decreased ubiquitination have been shown to improve apoB lipidation thereby increasing VLDL size. We observed no diet differences in plasma apoB, *i.e.*, VLDL particle number, consistent with results in other animal models, including non-human primates [59,60]. When cultured rat hepatocytes were incubated with either EPA or DHA, VLDL secretion was impaired in comparison to oleic acid [61]. Furthermore, DHA incubation resulted in less TG secretion *vs*. EPA. This may partially explain why FO, but not EO, resulted in decreased TG secretion compared to PO in our studies.

FO reduces plasma TG by reducing lipogenesis; however, other studies have suggested increased TG lipolysis and removal by FO as another potential mechanism. Our turnover studies in PO- and EO-fed mice showed a subtle increase in the removal of EO VLDL particles from plasma. A study of short-term (3–5 weeks) FO feeding in humans reported an increased fractional catabolic rate for VLDL-TG, and possibly decreased VLDL size [50]. Decreased VLDL size may be a function of increased lipase activity; however, whether increased lipolysis is proportional to lipase activity is unclear [45,62]. PUFA-enriched chylomicrons are better substrates for lipoprotein lipase (LPL) than those containing SFAs *in vitro* [63], suggesting increased enzyme-substrate kinetics with FO. Our VLDL chemical analysis showed that *n*-3 PUFA enrichment decreased VLDL particle size. We found that altered VLDL size and composition resulted in changes in lipolysis susceptibility. Lipase activity was not altered among the three groups, but VLDL from EO- and FO-fed mice was more easily hydrolyzed. These findings show that cell enrichment with *n*-3 PUFAs can alter the physical properties of lipoproteins, allowing more efficient substrate-enzyme kinetics.

## 5. Conclusions

In summary, we have shown that plasma TG concentrations were reduced in EO- and FO-fed apoB100-only LDLrKO mice compared to those fed PO. However, TG lowering by FO and EO did not occur by parallel mechanisms. FO feeding appears to lower plasma TG by reducing hepatic TG secretion and increasing VLDL hydrolysis susceptibility. Alternatively, EO feeding appears to lower plasma TG primarily by increasing intravascular lipolysis, with no effect on hepatic TG secretion. While EO reduced plasma TG concentrations, it did not prevent hepatic steatosis in this mouse model, contrary to FO feeding. Therefore, DHA, which is enriched in lipid fractions with FO, but not EO, feeding, probably plays a greater role in reducing hepatic TG levels than EPA, which is enriched with both FO and EO feeding. Further studies are necessary to completely understand the contributions of EPA and DHA to hepatic lipid metabolism. Our studies suggest that EO possesses cardioprotective properties comparable to those of FO [64,65]. Though further studies are needed to determine possible adverse effects of EO, such as fatty liver, our findings preliminarily suggest that EO may be an alternative to FO for treating CVD, particular in subjects who are FO intolerant.

## Supplementary Files

Supplementary File 1 Supplementary (DOCX, 35 KB)

## References

[1] Defilippis A.P., Blaha M.J., Jacobson T.A. (2010). Omega-3 fatty acids for cardiovascular disease prevention. Curr. Treat. Options Cardiovasc. Med..

[2] Kromhout D., Giltay E.J., Geleijnse J.M. (2010). *N*-3 fatty acids and cardiovascular events after myocardial infarction. N. Engl. J. Med..

[3] Roth E.M., Harris W.S. (2010). Fish oil for primary and secondary prevention of coronary heart disease. Curr. Atheroscler. Rep..

[4] Dyerberg J., Bang H.O. (1982). A hypothesis on the development of acute myocardial infarction in Greenlanders. Scand. J. Clin. Lab. Investig..

[5] Dyerberg J., Bang H.O. (1979). Lipid metabolism, atherogenesis, and haemostasis in Eskimos: The role of the prostaglandin-3 family. Haemostasis.

[6] Bang H.O., Dyerberg J., Hjoorne N. (1976). The composition of food consumed by Greenland Eskimos. Acta Med. Scand..

[7] Davis H.R., Bridenstine R.T., Vesselinovitch D., Wissler R.W. (1987). Fish oil inhibits development of atherosclerosis in rhesus monkeys. Arteriosclerosis.

[8] Rudel L.L., Kelley K., Sawyer J.K., Shah R., Wilson M.D. (1998). Dietary monounsaturated fatty acids promote aortic atherosclerosis in LDL receptor-null, human ApoB100-overexpressing transgenic mice. Arterioscler. Thromb. Vasc. Biol..

[9] Parks J.S., Kaduck-Sawyer J., Bullock B.C., Rudel L.L. (1990). Effect of dietary fish oil on coronary artery and aortic atherosclerosis in African green monkeys. Arteriosclerosis.

[10] Albert C.M., Campos H., Stampfer M.J., Ridker P.M., Manson J.E., Willett W.C., Ma J. (2002). Blood levels of long-chain *n*-3 fatty acids and the risk of sudden death. N. Engl. J. Med..

[11] Burr M.L., Fehily A.M., Gilbert J.F., Rogers S., Holliday R.M., Sweetnam P.M., Elwood P.C., Deadman N.M. (1989). Effects of changes in fat, fish, and fibre intakes on death and myocardial reinfarction: Diet and reinfarction trial (DART). Lancet.

[12] Sanders T.A., Sullivan D.R., Reeve J., Thompson G.R. (1985). Triglyceride-lowering effect of marine polyunsaturates in patients with hypertriglyceridemia. Arteriosclerosis.

[13] Harris W.S., Connor W.E., Alam N., Illingworth D.R. (1988). Reduction of postprandial triglyceridemia in humans by dietary *n*-3 fatty acids. J. Lipid Res..

[14] Norling L.V., Serhan C.N. (2010). Profiling in resolving inflammatory exudates identifies novel anti-inflammatory and pro-resolving mediators and signals for termination. J. Intern. Med..

[15] Harris W.S. (1989). Fish oils and plasma lipid and lipoprotein metabolism in humans: A critical review. J. Lipid Res..

[16] Harris W.S. (1996). *N*-3 fatty acids and lipoproteins: Comparison of results from human and animal studies. Lipids.

[17] Kris-Etherton P.M., Yu S. (1997). Individual fatty acid effects on plasma lipids and lipoproteins: Human studies. Am. J. Clin. Nutr..

[18] Cullen P. (2000). Evidence that triglycerides are an independent coronary heart disease risk factor. Am. J. Cardiol..

[19] Oosterveer M.H., van Dijk T.H., Tietge U.J., Boer T., Havinga R., Stellaard F., Groen A.K., Kuipers F., Reijngoud D.J. (2009). High fat feeding induces hepatic fatty acid elongation in mice. PLoSOne.

[20] Larter C.Z., Yeh M.M., Cheng J., Williams J., Brown S., dela Pena A., Bell-Anderson K.S., Farrell G.C. (2008). Activation of peroxisome proliferator-activated receptor alpha by dietary fish oil attenuates steatosis, but does not prevent experimental steatohepatitis because of hepatic lipoperoxide accumulation. J. Gastroenterol. Hepatol..

[21] Jump D.B., Clarke S.D. (1999). Regulation of gene expression by dietary fat. Annu. Rev. Nutr..

[22] Vasandani C., Kafrouni A.I., Caronna A., Bashmakov Y., Gotthardt M., Horton J.D., Spady D.K. (2002). Upregulation of hepatic LDL transport by *n*-3 fatty acids in LDL receptor knockout mice. J. Lipid Res..

[23] Qi K., Fan C., Jiang J., Zhu H., Jiao H., Meng Q., Deckelbaum R.J. (2008). Omega-3 fatty acid containing diets decrease plasma triglyceride concentrations in mice by reducing endogenous triglyceride synthesis and enhancing the blood clearance of triglyceride-rich particles. Clin. Nutr..

[24] Wang H., Chen X., Fisher E.A. (1993). *N*-3 fatty acids stimulate intracellular degradation of apoprotein B in rat hepatocytes. J. Clin. Investig..

[25] Halvorsen B., Rustan A.C., Madsen L., Reseland J., Berge R.K., Sletnes P., Christiansen E.N. (2001). Effects of long-chain monounsaturated and *n*-3 fatty acids on fatty acid oxidation and lipid composition in rats. Ann. Nutr. Metab..

[26] Kris-Etherton P.M., Taylor D.S., Yu-Poth S., Huth P., Moriarty K., Fishell V., Hargrove R.L., Zhao G., Etherton T.D. (2000). Polyunsaturated fatty acids in the food chain in the United States. Am. J. Clin. Nutr..

[27] Burdge G. (2004). α-Linolenic acid metabolism in men and women: Nutritional and biological implications. Curr. Opin. Clin. Nutr. Metab. Care.

[28] Nakamura M.T., Nara T.Y. (2004). Structure, function, and dietary regulation of delta6, delta5, and delta9 desaturases. Annu. Rev. Nutr..

[29] Guil-Guerrero J.L., Gomez-Mercado F., Garcia-Maroto F., Campra-Madrid P. (2000). Occurrence and characterization of oils rich in gamma-linolenic acid Part I: Echium seeds from Macaronesia. Phytochemistry.

[30] Zhang P., Boudyguina E., Wilson M.D., Gebre A.K., Parks J.S. (2008). Echium oil reduces plasma lipids and hepatic lipogenic gene expression in apoB100-only LDL receptor knockout mice. J. Nutr. Biochem..

[31] Powell-Braxton L., Veniant M., Latvala R.D., Hirano K.I., Won W.B., Ross J., Dybdal N., Zlot C.H., Young S.G., Davidson N.O. (1998). A mouse model of human familial hypercholesterolemia: Markedly elevated low density lipoprotein cholesterol levels and severe atherosclerosis on a low-fat chow diet. Nat. Med..

[32] Farese R.V., Veniant M.M., Cham C.M., Flynn L.M., Pierotti V., Loring J.F., Traber M., Ruland S., Stokowski R.S., Huszar D. (1996). Phenotypic analysis of mice expressing exclusively apolipoprotein B48 or apolipoprotein B100. Proc. Natl. Acad. Sci. USA.

[33] Bell T.A., Kelley K., Wilson M.D., Sawyer J.K., Rudel L.L. (2007). Dietary fat-induced alterations in atherosclerosis are abolished by ACAT2-deficiency in apoB100 only, LDLr^−/−^ mice. Arterioscler. Thromb. Vasc. Biol..

[34] Furbee J.W., Francone O., Parks J.S. (2001). Alteration of plasma HDL cholesteryl ester composition with transgenic expression of a point mutation (E149A) of human LCAT. J. Lipid Res..

[35] Bligh E.G., Dyer W.J. (1959). A rapid method of total lipid extraction and purification. Can. J. Biochem. Physiol..

[36] Livak K.J., Schmittgen T.D. (2001). Analysis of relative gene expression data using real-time quantitative PCR and the 2(-Delta Delta C(T)) method. Methods.

[37] Rouser G., Fkeischer S., Yamamoto A. (1970). Two dimensional then layer chromatographic separation of polar lipids and determination of phospholipids by phosphorus analysis of spots. Lipids.

[38] Lee J.Y., Timmins J.M., Mulya A., Smith T.L., Zhu Y., Rubin E.M., Chisholm J.W., Colvin P.L., Parks J.S. (2005). HDLs in apoA-I transgenic Abca1 knockout mice are remodeled normally in plasma but are hypercatabolized by the kidney. J. Lipid Res..

[39] Wilcox R.W., Thuren T., Sisson P., Kucera G.L., Waite M. (1991). Hydrolysis of neutral lipid substrates by rat hepatic lipase. Lipids.

[40] Otway S., Robinson D.S. (1967). The use of a non-ionic detergent (Triton WR 1339) to determine rates of triglyceride entry into the circulation of the rat under different physiological conditions. J. Physiol..

[41] McFarlane A.S. (1958). Efficient trace-labelling of proteins with iodine. Nature.

[42] Lee J.Y., Lanningham-Foster L., Boudyguina E.Y., Smith T.L., Young E.R., Colvin P.L., Thomas M.J., Parks J.S. (2004). Preβ high density lipoprotein has two metabolic fates in human apolipoprotein A-I transgenic mice. J. Lipid Res..

[43] Davidson M.H. (2006). Mechanisms for the hypotriglyceridemic effect of marine omega-3 fatty acids. Am. J. Cardiol..

[44] Qi K., Al-Haideri M., Seo T., Carpentier Y.A., Deckelbaum R.J. (2003). Effects of particle size on blood clearance and tissue uptake of lipid emulsions with different triglyceride compositions. J. Parenter. Enter. Nutr..

[45] Nozaki S., Garg A., Vega G.L., Grundy S.M. (1991). Postheparin lipolytic activity and plasma lipoprotein response to omega-3 polyunsaturated fatty acids in patients with primary hypertriglyceridemia. Am. J. Clin. Nutr..

[46] Egusa G., Brady D.W., Grundy S.M., Howard B.V. (1983). Isopropanol precipitation method for the determination of apolipoprotein B specific activity and plasma concentrations during metabolic studies of very low density lipoprotein and low density lipoprotein apolipoprotein B. J. Lipid Res..

[47] Ishibashi S., Brown M.S., Goldstein J.L., Gerard R.D., Hammer R.E., Herz J. (1993). Hypercholesterolemia in low density lipoprotein receptor knockout mice and its reversal by adenovirus-mediated gene delivery. J. Clin. Investig..

[48] Surette M.E., Edens M., Chilton F.H., Tramposch K.M. (2004). Dietary echium oil increases plasma and neutrophil long-chain (*n*-3) fatty acids and lowers serum triacylglycerols in hypertriglyceridemic humans. J. Nutr..

[49] Bordin P., Bodamer O.A., Venkatesan S., Gray R.M., Bannister P.A., Halliday D. (1998). Effects of fish oil supplementation on apolipoprotein B100 production and lipoprotein metabolism in normolipidaemic males. Eur. J. Clin. Nutr..

[50] Harris W.S., Connor W.E., Illingworth D.R., Rothrock D.W., Foster D.M. (1990). Effects of fish oil on VLDL triglyceride kinetics in humans. J. Lipid Res..

[51] Nestel P.J., Connor W.E., Reardon M.F., Connor S., Wong S., Boston R. (1984). Suppression by diets rich in fish oil of very low density lipoprotein production in man. J. Clin. Investig..

[52] Parks J.S., Johnson F.L., Wilson M.D., Rudel L.L. (1990). Effect of fish oil diet on hepatic lipid metabolism in nonhuman primates: Lowering of secretion of hepatic triglyceride but not apoB. J. Lipid Res..

[53] Nestel P.J. (2000). Fish oil and cardiovascular disease: Lipids and arterial function. Am. J. Clin. Nutr..

[54] Jump D.B. (2004). Fatty acid regulation of gene transcription. Crit. Rev. Clin. Lab. Sci..

[55] Xu J., Nakamura M.T., Cho H.P., Clarke S.D. (1999). Sterol regulatory element binding protein-1 expression is suppressed by dietary polyunsaturated fatty acids. A mechanism for the coordinate suppression of lipogenic genes by polyunsaturated fats. J. Biol. Chem..

[56] Kim H.J., Takahashi M., Ezaki O. (1999). Fish oil feeding decreases mature sterol regulatory element-binding protein 1 (SREBP-1) by down-regulation of SREBP-1c mRNA in mouse liver. A possible mechanism for down-regulation of lipogenic enzyme mRNAs. J. Biol. Chem..

[57] Yahagi N., Shimano H., Hasty A.H., Amemiya-Kudo M., Okazaki H., Tamura Y., Iizuka Y., Shionoiri F., Ohashi K., Osuga J. (1999). A crucial role of sterol regulatory element-binding protein-1 in the regulation of lipogenic gene expression by polyunsaturated fatty acids. J. Biol. Chem..

[58] Pan M., Cederbaum A.I., Zhang Y.L., Ginsberg H.N., Williams K.J., Fisher E.A. (2004). Lipid peroxidation and oxidant stress regulate hepatic apolipoprotein B degradation and VLDL production. J. Clin. Investig..

[59] Wong S.H., Nestel P.J., Trimble R.P., Stoker G.B., Illman R.J., Topping D.L. (1984). The adaptive effects of dietary fish and safflower oil on lipid and lipoprotein metabolism in perfused rat liver. Biochim. Biophys. Acta.

[60] Parks J.S., Wilson M.D., Johnson F.L., Rudel L.L. (1989). Fish oil decreases hepatic cholesteryl ester secretion but not apoB secretion in African green monkeys. J. Lipid Res..

[61] Lang C.A., Davis R.A. (1990). Fish oil fatty acids impair VLDL assembly and/or secretion by cultured rat hepatocytes. J. Lipid Res..

[62] Hulsmann W.C., Oerlemans M.C., Jansen H. (1980). Activity of heparin-releasable liver lipase. Dependence on the degree of saturation of the fatty acids in the acylglycerol substrates. Biochim. Biophys. Acta.

[63] Weintraub M.S., Zechner R., Brown A., Eisenberg S., Breslow J.L. (1988). Dietary polyunsaturated fats of the W-6 and W-3 series reduce postprandial lipoprotein levels. Chronic and acute effects of fat saturation on postprandial lipoprotein metabolism. J. Clin. Investig..

[64] Forrest L.M., Boudyguina E., Wilson M.D., Parks J.S. (2012). Echium oil reduces atherosclerosis in apoB100-only LDLrKO mice. Atherosclerosis.

[65] Brown A.L., Zhu X., Rong S., Shewale S., Seo J., Boudyguina E., Gebre A.K., Alexander-Miller M.A., Parks J.S. (2012). Omega-3 fatty acids ameliorate atherosclerosis by favorably altering monocyte subsets and limiting monocyte recruitment to aortic lesions. Arterioscler. Thromb. Vasc. Biol..

