# Supplementary material for: Echium Oil Reduces Plasma Triglycerides by Increasing Intravascular Lipolysis in apoB100-Only Low Density Lipoprotein (LDL) Receptor Knockout Mice
Source: Nutrients. 2013 Jul 12;5(7):2629–45. doi: 10.3390/nu5072629 (PMC3738992; doi:10.3390/nu5072629)
Supplement: Supplementary File 1 — Supplementary (DOCX, 35 KB) [file nutrients-05-02629-s001.docx]

**Supplemental Data**

**1. Experimental Section**

**Dietary oils:** The seed oil of *Echium plantagineum* L., a member of the Boraginaceae family was a generous gift from Croda Europe Ltd. (Leek, Staffordshire, UK) and authenticated by the Wake Forest University Center for Botanical Lipids and Inflammatory Disease Prevention. A certificate of analysis is on file for reference along with retention samples deposited at the Wake Forest School of Medicine. The seed oil of palm, *Elaeis guineensis* Jacq*.*, a member of the Arecaceae family, was purchased from Shay and Company (Portland, OR, USA). A certificate of analysis is on file for reference. The fish oil source was *Brevoortia tyrannis* Latrobe, a member of the Clupeidae family, was manufactured and generously provided by Omega Protein (Reidsville, VA, USA) with a report of analysis on file for reference.

**2. Supplemental Figure**

**Figure S1.** Fatty acid composition of plasma lipids. After 16 weeks of PO, EO, or FO experimental diet feeding, plasma was collected for measurement of PL, TG, and CE fatty acid composition. Lipids from plasma were extracted using the Bligh-Dyer method and separated into PL, TG, and CE bands by thin layer chromatography. The bands were visualized with primuline and collected, after which fatty acids were transmethylated and analyzed for fatty acid distribution by gas-liquid chromatography as described in the Materials and Methods section. Data represent mean ± S.E.M.; *n* = 5 for each group. Values with different letters are significantly different (*p* < 0.05) by ANOVA; data bars not marked with letters were not significantly different. The figure only shows polyunsaturated fatty acids (≥2 double bonds).

© 2013 by the authors; licensee MDPI, Basel, Switzerland. This article is an open access article distributed under the terms and conditions of the Creative Commons Attribution license (http://creativecommons.org/licenses/by/3.0/).
